# Optimization of Enzymatic Hydrolysis and Fermentation Processing for Set-Type Oat Yogurt with Favorable Acidity and Coagulated Texture

**DOI:** 10.3390/foods13244180

**Published:** 2024-12-23

**Authors:** Wenjie Xu, Xinzhu Wu, Chen Xia, Zicong Guo, Zhengyuan Zhai, Yongqiang Cheng, Ju Qiu

**Affiliations:** 1 Key Laboratory of Precision Nutrition and Food Quality, Department of Nutrition and Health, China Agricultural University, No. 17 Tsinghua East Road, Haidian District, Beijing 100083, China; sy20223061356@cau.edu.cn (W.X.); xia453853384@163.com (C.X.); gzcjasmine@163.com (Z.G.); zhaizy@cau.edu.cn (Z.Z.); chengyq@cau.edu.cn (Y.C.); 2 Beijing Laboratory of Food Quality and Safety, College of Food Science and Nutritional Engineering, China Agricultural University, No. 17 Tsinghua East Road, Haidian District, Beijing 100083, China; 3 State Key Laboratory of Food Nutrition and Safety, College of Food Science and Engineering, Tianjin University of Science and Technology, Tianjin 300457, China; 18835431263@163.com

**Keywords:** oat yogurt, enzymatic hydrolysis, fermentation, exopolysaccharide, coagulated texture profile

## Abstract

The key role of enzymatic hydrolysis and fermentation in the sensory quality of set yogurt made from whole oats was demonstrated. The optimal process was established by the orthogonal and response surface methodology based on the acidity, textural, and rheological properties. The results indicated that the enzymatic hydrolysis appropriately consisted of liquefaction with 12 U/mL α-amylase at 70 °C and pH 6.5 for 60 min, followed by saccharification with 400 U/mL α-1,4-glucan glucohydrolase at 60 °C and pH 4.5 for 60 min. The *Streptococcus thermophilus ST15* and *Lactobacillus bulgaricus 20249* strains were the most efficacious strains, with a 0.1% inoculation for the fermentation at 42 °C for 16 h. So, a soft semisolid oat yogurt formed with an 8% solid–liquid ratio, which exhibited an acidity of 73.17 °T, a cohesiveness ratio of 0.51, and a maximum apparent viscosity of 1902.67 Pa·s. The coagulated texture of the oat yogurt was closely associated with the exopolysaccharide (EPS) yield up to 304.99 mg/L. These findings supported the optimal processing of oat yogurt, especially its correlation with the high capacity of EPS production by strains. It is an innovative and feasible way to improve the properties of set-type oat yogurt, especially the utilization of the whole oat.

## 1. Introduction

Traditional yogurt, made from milk fermented by lactic acid bacteria, has long been favored as a significant source of high-quality dietary protein [1]. However, yogurt consumption may lead to issues such as lactose intolerance, hypercholesterolemia, and milk allergy, rendering it unsuitable for certain individuals, including vegans [2]. More importantly, these challenges have hindered the further development of the yogurt industry. Plant-based yogurt has emerged as a potential solution to these problems [3]. Firstly, plant-based yogurt is lactose-free and contains no or small amounts of allergens, making it very friendly to people with lactose intolerance or milk allergies [4]. Secondly, plant-based yogurt offers cholesterol-free, low saturated lipids, and many bioactive compounds from plant ingredients, providing a suitable alternative for vegans or consumers with healthy diet requirements. Finally, plant-based yogurt is much friendlier for the environment because its production requires less carbon emissions and water pollution than animal yogurt, depending on breeding industry. The advent of plant-based yogurt effectively addresses the limitations inherent in traditional yogurt and catalyzes the evolution of the yogurt industry.

As a worldwide plant resource, oats are distinguished from other cereal crops by their relative higher protein content, which, in naked oat varieties, reaches up to 20%. This is the reason why oats are predominantly utilized in plant-protein beverages in addition to soybeans; for example, oat milk is currently appealing to increasing numbers of consumers [5]. Therefore, more and more research has become focused on fermented oat drinks, because of the different flavor and sensory qualities. Unlike soy or coconut yogurts, the development of oat yogurt faces significant hurdles due to its high content of starch and dietary fiber. There are several technical challenges for oat yogurt production, and it is especially difficult to produce set-type oat yogurt. Firstly, the primary carbohydrate in oats is starch, accounting for over 60%, rather than monosaccharides, limiting the acid production by lactic acid bacteria. Secondly, during fermentation and storage, insoluble components such as protein and dietary fiber in oats tend to aggregate and precipitate, causing stratification or flocculation in oat yogurt. Thirdly, oat protein mainly consists of gluten, making it difficult to form a stable gel-like casein, which cannot rely on protein coagulation to obtain a texture similar to animal milk yogurt [6]. These technical challenges have hindered the development of the oat yogurt processing industry, resulting in a lack of set-type oat yogurt products on the market until now. Set-type yogurt is a type of yogurt that is fermented directly in the container in which it is sold. It is noted that the production process of set-type yogurt is not only simpler than that of stirred yogurt, but also preserves the gel structure without disruption. It is thus essential to develop set-type oat yogurt products [7].

Several studies have been conducted to address the aforementioned problems of fermented oat drinks. Most studies have supplemented the necessary carbon source for lactic acid bacteria by adding exogenous sugars, such as glucose and fructose [8]. However, it fails to meet the demands of present consumers for “less sugar” intake or “no added sugar” in ingredient lists. Enzyme hydrolysis technology has been developed to enable the conversion of oat starch into monosaccharides, including glucose for subsequent fermentation [9]. Nevertheless, because of the insufficient enzymatic hydrolysis, the aggregation of insoluble proteins and dietary fibers leads to precipitate formation and its removal by filtration. Unfortunately, this filtration results in the loss of insoluble nutrients, thwarting the nutritional preservation of the whole oat. More importantly, it is impossible to produce set-type oat yogurt by using an enzymatic hydrolysate due to the lack of stickiness. Given the poor coagulability of oat protein, the exogenous additives of thickeners are used to increase the apparent viscosity of set-type yogurt in the process of fermentation [10]. Despite this, the stability and texture of set-type oat yogurt are still not satisfactory. The change in properties of oat starch gel in the process of fermentation remains unexplored. Moreover, although the exopolysaccharides (EPSs) produced by lactic acid bacteria are pivotal in enhancing the stability of animal milk yogurt [11], their impact on the stability and textural attributes of set-type oat yogurt has not been investigated.

This study innovatively solved the technical problems of set-type oat yogurt by composing an oat enzymatic hydrolysate as an endogenous carbon source, together with the whole oat flour as an endogenous coagulant. The process of enzymatic hydrolysis was firstly investigated by an orthogonal design based on the dextrose equivalent (DE) value of the hydrolysate to avoid the external addition of sugar in the final products of set-type oat yogurt. The process of the fermentation of the mixture of the enzymatic hydrolysate and the whole oat flour was investigated by the response surface methodology (RSM) based on acidity, pH value, cohesiveness, and apparent viscosity optimization. Furthermore, the improved mechanism of the optimal strain was further clarified by analyzing the key role of EPSs produced by lactic acid bacteria in the textural profile of set-type yogurt.

## 2. Methods and Materials

### 2.1. Materials

The oatmeal was purchased from Beijing Special Fat Lowering Oat Development Co., Ltd. (Beijing, China). According to the labeled composition, a 100 g portion of oatmeal was composed of 56.7 g of carbohydrates, 15.6 g of proteins, and 7.4 g of fats. The enzyme activity was determined as described by Wang et al. with slight modifications [12]. Herein, one unit of enzymatic activity was defined as the number of enzymes that catalyzed the production of 1 mg of reducing sugar per hour. The results were shown as units per gram (U/g). The activity of α-amylase obtained from Shanghai Macklin Biochemical Technology Co., Ltd. (Shanghai, China) was measured as 4098.67 U/g. The activity of α-1,4-glucan glucohydrolase from Shanghai Yuanye Bio-Technology Co., Ltd. (Shanghai, China) was measured as 101,055.33 U/g. *Streptococcus thermophilus ST15 (ST15)* was purchased from Probiotical SpA (Novara, Italy), and *Streptococcus thermophilus 20370 (ST20370)*, *Lactobacillus bulgaricus 20247 (LB20247)*, *Lactobacillus bulgaricus 20249 (LB20249)*, and *Lactobacillus bulgaricus 20271 (LB20271)* were purchased from China National Research Institute of Food and Fermentation Industries (Beijing, China). De Man–Rogosa–Sharpe, De Man–Rogosa–Sharpe Broth, and Modified Chalmers medium were purchased from Aoqi Kehua Medical Supply Chain Management Services Co., Ltd. (Tianjin, China). Sodium hydroxide, sodium chloride, phenolphthalein, sulphuric acid, trichloroacetic acid, and phenol were analytical reagents and purchased from Sinopharm Chemical Reagent Co., Ltd. (Beijing, China).

### 2.2. Preparation of Oat Yogurt

#### 2.2.1. Enzymatic Hydrolysis of Oat Flour

Oatmeal was ground into an 80-mesh fine powder, and 10% of oat flour was mixed with 90% of water to prepare the oat slurry. An orthogonal design was used to investigate the process of enzymatic hydrolysis. Concretely, a certain amount of α-amylase (4, 8, 12, 16, 20 U/mL) was added into the oat slurry to trigger the enzymatic hydrolysis reaction at different temperatures (60, 65, 70, 75, 80 °C), times (30, 40, 50, 60, 70 min), and pH values (5.0, 5.5, 6.0, 6.5, 7.0). The temperature was raised to 95 °C to inactivate the enzyme for 10 min, followed by cooling down to room temperature. Next, α-1,4-glucan glucohydrolase (100, 200, 300, 400, 500 U/mL) was added at different temperatures (50, 55, 60, 65, 70 °C), times (30, 40, 50, 60, 70 min), and pH values (3.5, 4.0, 4.5, 5.0, 5.5), followed by the inactivation at 95 °C for 10 min. The enzymatic hydrolysate of oats was ready for the determination of DE values and subsequent oat yogurt preparation.

#### 2.2.2. Heating of Oat Hydrolysate and Oat Flour Mixture

The oat enzymatic hydrolysate was mixed with the oat flour with different proportions of 5, 7, 9, 11, and 13%, followed by the heating at 100 °C for 15 min. A uniform oat paste was obtained by homogenization and cooled down to 45 °C for the subsequent fermentation.

#### 2.2.3. Fermentation and Ripening of Oat Yogurt

A mixed bacterial solution of *Streptococcus thermophilus* and *Lactobacillus bulgaricus* with equal amounts was inoculated to the oat paste at different doses (0.001, 0.025, 0.050, 0.075, 0.100%). After stirring evenly, it was fermented at different temperatures (32, 37, 42, 47, 52 °C) and times (8, 12, 16, 20, 24 h). The fermented yogurt was stored at 4 °C in a refrigerator for 24 h to allow for maturation. The enzymatic hydrolysate of oats was fermented directly without oat flour in order to be used as a control (fermented oat hydrolysate).

### 2.3. Experimental Optimization Design

The RSM is a systematic mathematical model for evaluating the relative importance and interaction of each parameter (inoculation volume, solid–liquid ratio, and temperature). To obtain a high-quality oat yogurt, the RSM based on a three-factor and three-level Box–Behnken design (BBD) was employed to study the optimal fermentation conditions for oat yogurt. The values of acidity, pH, cohesiveness, and apparent viscosity of the oat yogurt were obtained based on the single-factor experiment. The objectives for acidity and cohesiveness were set to be maximized, while the objective for pH was set to be minimized. The apparent viscosity of Sanyuan set-type yogurt, which is the ideal apparent viscosity reference for oat yogurt, was 1889 Pa·s. This experimental design consisted of 17 runs in total, including 12 factor points and 5 replications at the center point to estimate the experimental error. The response factors were monitored with a second-order polynomial equation (Equation (1)) to figure out the predicted response (Y).

Y = β_0_ + β_1_A + β_2_B + β_3_C + β_12_AB + β_13_AC + β_23_BC + β_11_A^2^ + β_22_B^2^ + β_33_C^2^ (1)

where Y is the response variable (sensory score); β_0_ is a constant; β_1_ and β_2_ are the linear coefficients; β_11_, β_22_, and β_33_ are the quadratic coefficients; β_12_, β_13_, and β_23_ are the interaction coefficients. A, B, and C represent inoculation volume, solid–liquid ratio, and temperature, respectively. The BBD output also contains the 3D surface response plot, which indicates the interaction between the responses and independent variables.

### 2.4. Determination of DE Value of Enzymatic Hydrolysate

The DE value of the oat hydrolysate described the extent to which starch was converted into reducing sugars during the hydrolysis process. It was quantified using the 3,5-dinitrosalicylic acid (DNS) method and expressed as a percentage relative to glucose on a dry basis [13]. Approximately 1 g of the oat hydrolysate was diluted to 50 mL and centrifuged to obtain the supernatant. The supernatant was then mixed with 3,5-dinitrosalicylic acid to measure the content of reducing sugar. DE value was calculated using the following equation:

DE value = reducing sugar content × 100%/total solid content (2)

### 2.5. Water-Holding Capacity (WHC)

The WHC was determined as described by Raikos et al. with slight modifications [14]. Briefly, 20 g of oat yogurt (Y) was placed in a 50 mL tube and centrifuged at 10,000 rpm for 20 min at 4 °C using a centrifuge (Avanti JXN-26; Beckman Coulter, Pasadena, CA, USA). The residue was collected and weighed (W). The WHC was calculated using the following equation:

WHC (%) = W/Y × 100% (3)

### 2.6. Determination of pH Value and Titratable Acidity of Oat Yogurt

The pH value and titratable acidity were determined as described by Kalyas et al. with slight modifications [15]. The pH value of oat yogurt was measured using a pH meter (S220-uMix; Mettler Toledo, Zurich, Switzerland) at room temperature. Titratable acidity was determined by titrating oat yogurt with 0.1 N NaOH, and phenolphthalein was used as an indicator. All samples were measured in triplicate.

### 2.7. Microbiological Determination of Oat Yogurt

The total viable bacterial counts of *Lactobacillus bulgaricus* and *Streptococcus thermophilus* were measured in accordance with the national food safety standard (GB 4789.35-2023; National food safety standard: Food microbiological examination: Examination of lactic acid bacteria. National Health Commission of the People’s Republic of China, and State Administration for Market Regulation of the People’s Republic of China: Beijing, China, 2023.) [16]. The oat yogurt was diluted to the required concentration in sterilized dilution buffer. *Lactobacillus bulgaricus* strains were detached using the De Man–Rogosa–Sharpe medium and cultured at 37 °C in an anaerobic atmosphere for 72 h. *Streptococcus thermophilus* strains were cultured at 37 °C in a Modified Chalmers medium under the aerobic culture environment for 72 h. Plates containing 30–300 colonies were selected to count the colonies (CFU/g).

### 2.8. Texture Profile Analysis of Oat Yogurt

The textural properties of oat yogurt were measured using a Texture Analyzer (TMS-Pro; Sterling, VA, USA) equipped with a 25 mm diameter cylindrical probe. The loadcell range was set as 0.2 N, the deformation percentage was 30, and the test speed was 50 mm/min.

### 2.9. Steady Shear Rheological Analysis of Oat Yogurt

The steady shear rheological properties of oat yogurt were determined using an AR-1500EX Rheometer (Ta Instruments-Waters LLC, Newcastle, DE, USA). To obtain steady shear data, a plate/plate geometry (40 mm diameter, 0.5 mm gap) was used at 25 °C with a shear rate of 0.01–100/s. The reported results were expressed as the average of three measurements.

### 2.10. Dynamic Shear Rheological Analysis of Oat Yogurt

The dynamic shear rheological properties [storage modulus (G′), loss modulus (G″)] of oat yogurt were determined using a strain-controlled AR-1500EX Rheometer (Ta Instruments-Waters LLC, Newcastle, DE, USA). A strain sweep test at a constant frequency determined the linear viscoelastic region. The dynamic shear rheological properties were then measured at a strain value of 0.5 (within the linear viscoelastic region). Frequency sweep tests were detected using a plate/plate geometry (40 mm diameter, 0.5 mm gap) at 25 °C and frequency of 0.1–10 Hz. The results were expressed as the average of three measurements.

### 2.11. Quantification of EPSs

Strains were inoculated in De Man–Rogosa–Sharpe broth and aerobic fermented at 37 °C for 16 h. The fermentation broth was heated at 100 °C for 20 min; then, bacteria and denatured proteins were removed by centrifugation at 10,000 rpm for 20 min. Trichloroacetic acid (4%, *w*/*v*) was added to the supernatant and kept at 4 °C for 12 h, and protein removal by centrifugation was conducted. EPS was precipitated by adding the triple volume of cold absolute ethanol and staying overnight at 4 °C. The crude EPS was obtained by centrifugation at 10,000× *g* for 20 min to collect the precipitate, which was then dialyzed in distilled water at 4 °C for 24 h (molecular weight cut-off: 8000 Da. The amount of EPS produced by strains was determined according to a spectrophotometric phenol–sulfuric acid method. The absorbance at 490 nm was measured using a multi-mode reader (SYNERGY HTX; BioTek Instruments, Inc., Winooski, VT, USA), and the EPS concentration was expressed as the glucose equivalent.

### 2.12. Electronic Tongue Analysis

The sensory characteristics of oat yogurt were measured using the TS-5000Z E-tongue instrument (Insent Inc., Atsugi-shi, Japan), including sourness, saltiness, richness, umami, aftertaste-A, aftertaste-B, astringency, and bitterness. The oat yogurt was diluted with an equal volume of deionized water, followed by the centrifugation at 10,000 rpm/min for 10 min, and the supernatant was assayed in triplicate.

### 2.13. Statistical Analysis

All results were indicated as mean ± standard deviation (SD) for triplicate measurements, and the entire experimental process was repeated by independent hydrolysis or fermentation. Results were analyzed using SPSS 25.0 (SSPS Institute, Chicago, IL, USA). An independent *t*-test was performed to compare two groups, while a one-way analysis of variance (ANOVA) was used to compare three or more groups. All statistical significance levels were judged using the Least Significant Difference Test and/or Tukey’s values at *p* < 0.05. The experimental optimization design was analyzed by RSM with BBD, using Design-Expert version 13.0.15 (Statease Inc., Minneapolis, MN, USA). A two-tailed Pearson correlation analysis was used to analyze the statistical correlations among the variables. The principal component analysis (PCA) results were also presented as a variables map. All the figures were generated using Origin Pro 2024 (Origin-Lab, Northampton, MA, USA).

## 3. Results and Discussions

### 3.1. Effect of Enzymatic Hydrolysis on the DE Value of Oat Hydrolysate

**Figure 1:**
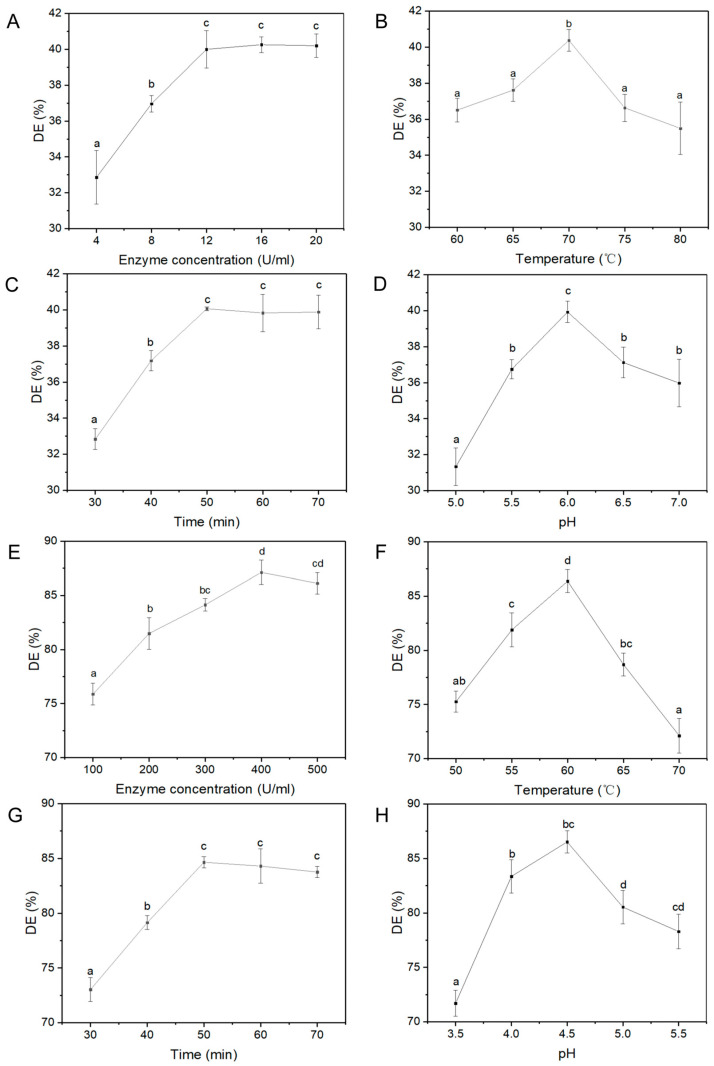
Changes in dextrose equivalent (DE) value of enzymatic hydrolysate under different liquefaction ((**A**) enzyme concentration; (**B**) temperature; (**C**) time; (**D**) pH) or saccharification conditions ((**E**) enzyme concentration; (**F**) temperature; (**G**) time; (**H**) pH). Lowercase letters expressed the statistical significance among different yogurt groups at *p* < 0.05.

Yogurt made of animal milk needs to be fermented by *Streptococcus thermophilus* and *Lactobacillus bulgaricus*, which consume lactose to generate lactic acid, resulting in increased acidity [17]. However, the present yogurt made of oats was so different from animal yogurt because there is no lactose in oats, but only oat starch and a few monosaccharides. Hence, it was crucial to supply the carbon source for further fermentation by the conversion from oat starch to monosaccharides, which relied on α-amylase and α-1,4-glucan glucohydrolase. The extent of the hydrolysis was reflected as the DE value. As the amount of α-amylase added increased, the DE value gradually increased and then stabilized, reaching the highest value of 40.00% at the dose of 12 U/mL enzyme (*p* < 0.05) (Figure 1A). The DE value initially increased with an increase in temperature but decreased from 70 °C with the highest value of 40.37% (*p* < 0.05) (Figure 1B). The DE value also gradually increased with a longer hydrolysis duration, which reached a plateau after 50 min, with the highest value of 40.06% (*p* < 0.05) (Figure 1C). As the same as the effects of temperature, the DE value initially increased with an increase in pH value but decreased then, peaking at pH value of 6.0 with the DE value of 39.93 % (*p* < 0.05) (Figure 1D). The influence of these conditions was as follows: temperature > pH > enzyme dose > time, according to the identification by the A2B2C3D3 combination through the K value (Table 1). The optimal liquefaction was an enzyme of 12 U/mL acting at 70 °C and a pH value of 6.5 for 60 min, which obtained the highest DE value of 43.56%.

As the amount of α-1,4-glucan glucohydrolase increased, the DE value gradually rose before stabilizing, reaching a maximum of 87.12% at a dose of 400 U/mL (*p* < 0.05) (Figure 1E). The DE value increased with rising temperature, peaking at 86.38% and then declining at temperatures above 60 °C (*p* < 0.05) (Figure 1F). Additionally, an extended duration of hydrolysis led to a gradual increase in the DE value, which eventually plateaued after 50 min, reaching a maximum of 84.65% (*p* < 0.05) (Figure 1G). The DE value initially increased with an increase in pH but decreased from 4.5 with the highest value of 86.51% (*p* < 0.05) (Figure 1H). The influence of α-1,4-glucan glucohydrolase was as follows: temperature > pH > enzyme dose > time, according to the identification by the A2B2C3D2 combination (Table 2). The optimal liquefaction was an enzyme of 400 U/mL acting at 60 °C and a pH value of 4.5 for 60 min, which obtained the highest DE value of 88.85%. Thus, the oat hydrolysate obtained through the optimal process was used for the subsequent set-type oat yogurt production.

### 3.2. Influence of Oat Flour on the Stability of Set-Type Oat Yogurt

**Figure 2:**
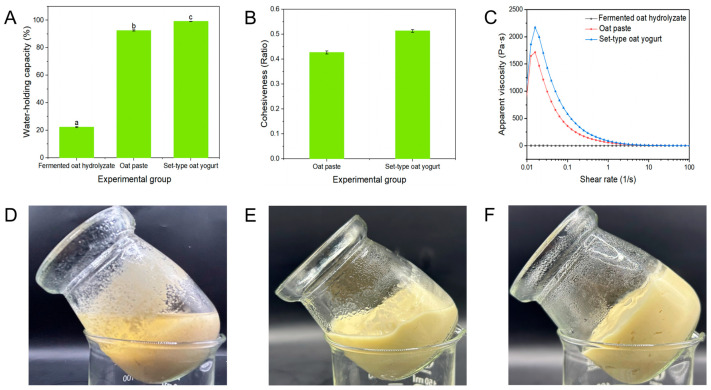
Water-holding capacity (**A**), cohesiveness (**B**), and viscosity (**C**) of fermented oat hydrolysate (**D**), oat paste (**E**), and set-type oat yogurt (**F**). For the fermented oat hydrolysate, the enzymatic hydrolysate of oats was fermented directly; for the oat paste, the enzymatic hydrolysate and flour mixture was heated; for the set-type oat yogurt, oat paste was fermented. Lowercase letters in the column express the statistical significance among different yogurt groups at *p* < 0.05.

Lactic acid bacteria produce acid as they grow and multiply in animal milk, which leads to the coagulation of casein at its isoelectric point so that the coagulated structure of set-type yogurt can form [18]. However, the oat protein was so different from casein because there were globulins and prolamins. Oat was more difficult to form into a protein gel compared to animal dairy, due to the different properties of the proteins [10]. Therefore, the present study developed an innovative way to produce the coagulated structure of set-type oat yogurt by starch gel. The WHC refers to the ability of a starch gel to maintain its liquid component, which directly impacts consumer satisfaction [19]. Yogurt with 1% of flaxseed powder exhibited a stronger WHC compared with yogurt without added flaxseed powder, increasing from 28.90% to 50.75% [15]. The WHC of oat paste (92.53%) was higher than that of fermented oat hydrolysate (22.27%) (*p* < 0.05) (Figure 2A), suggesting that the gelatinization of oat starch enabled the long and branched molecules of starch to readily bond with water, effectively minimizing the dehydration in the set-type oat yogurt [20]. The interaction between starch molecules and water was supported by the cohesiveness results (Figure 2B). The cohesiveness value of fermented oat hydrolysate could not be measured because of the excessive thinness and apparent layering, but the oat paste showed a high cohesiveness ratio value of 0.43. It is worth noting that the cohesiveness ratio of set-type oat yogurt was 0.5, which was higher than that of oat paste (*p* < 0.05). This increase might be attributed to the generation of EPSs by lactic acid bacteria in the fermentation process. EPSs were bacterial polysaccharides that could either be attached to cells (capsular EPS) or secreted into the extracellular medium (free EPSs) [21]. It increased the viscosity and consequently mitigated the layering phenomenon in the set-type oat yogurt. The EPS has been also reported to enhance the viscosity of animal yogurt by Nikitina et al. [22]. The subsequent determination of apparent viscosity also supported the key role of oat starch in heated oat paste in stabilizing the coagulated structure of set-type oat yogurt, because the apparent viscosity of fermented oat hydrolysate (3 Pa·s) was much lower than that of oat paste (1716 Pa·s) (*p* < 0.05) (Figure 2C). That was the reason why the enzymatic hydrolysate of oats was mixed with oat flour to produce oat paste and then fermented to obtain the set-type oat yogurt. It was impossible to obtain the coagulated structure by only the fermentation of oat hydrolysate, which showed serious layering (Figure 2D). The oat paste, which mainly consists of gelatinized starch, was necessary for the stable set-type yogurt (Figure 2E). The set-type oat yogurt in this study was stable without being watery or layered (Figure 2F).

### 3.3. Influence of Culture Strains on the Quality of Set-Type Oat Yogurt

**Figure 3:**
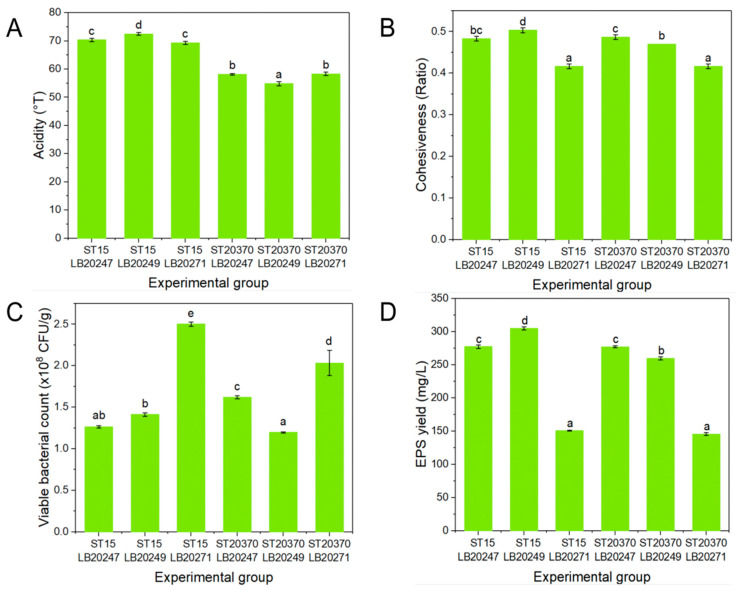
Acidity (**A**), cohesiveness (**B**), and viable bacterial count (**C**) of set-type oat yogurt fermented by different strain combinations, as well as the ability of these combinations to produce exopolysaccharides (EPSs) (**D**). *ST15*, *Streptococcus thermophilus15*; *ST20370*, *Streptococcus thermophilus 20370*; *LB20247*, *Lactobacillus bulgaricus 20247*; *LB20249*, *Lactobacillus bulgaricus 20249*; *LB20271*, *Lactobacillus bulgaricus 20271*. Lowercase letters in the column express the statistical significance among different yogurt groups at *p* < 0.05.

*Streptococcus thermophilus* and *Lactobacillus bulgaricus* are the commonest culture strains in the process of milk yogurt manufacturing for their acid and aroma production capacity [17]. In this study, different strains of *Streptococcus thermophilus* and *Lactobacillus bulgaricus* were used for the fermentation of set-type oat yogurt. The set-type oat yogurt fermented by *ST15* and *LB20249* exhibited the highest acidity (72.50 °T) (*p* < 0.05), which might be attributed to their having the best ability to utilize the glucose hydrolyzed from oat starch for lactic acid production (Figure 3A). The cohesiveness of set-type oat yogurt fermented with *ST15* and *LB20249* also exhibited the highest value up to a 0.50 ratio (Figure 3B). The viable bacterial count refers to the total quantity of active probiotics per unit weight or volume under specific conditions. In accordance with the GB 19302-2010 (GB 19302-2010; National Food Safety Standard Fermented Milk. Ministry of Health of the People’s Republic of China: Beijing, China, 2010.), the number of lactic acid bacteria in yogurt was required to be at least 10^6^ CFU/mL (g) [23]. In this study, the number of viable bacteria in each group of set-type oat yogurt met the requirements of the national standards (Figure 3C). EPS is an eco-friendly and non-toxic biopolymeric material that is widely used in various industrial fields such as pharmaceuticals, food, and cosmetics, due to its structural, rheological, and physicochemical properties [24]. A comparison of the EPS yield among different strains revealed that there was the highest output in the *ST15* and *LB20249* combination, producing 304.99 mg/L of EPS (*p* < 0.05) (Figure 3D). EPS played a crucial role in enhancing the coagulated structure and stability of the yogurt. Therefore, *ST15* and *LB20249* were utilized as the primary strains for the fermentation of set-type oat yogurt in the subsequent experiments.

As mentioned above, the coagulated structure of set-type oat yogurt was better than oat paste (Figure 2E,F). The higher WHC (Figure 2A), cohesiveness (Figure 2B), and apparent viscosity (Figure 2C) values of set-type oat yogurt were supported by the EPS production in the process of fermentation. The *ST15* and *LB20249* strains contributed to the highest increase in EPS production, as well as an increase in the WHC from 92.53% of the oat paste to 99.3% of the set-type oat yogurt (*p* < 0.05) (Figure 2A). In comparison, the WHC of set-type oat yogurt made from the whole oats in this study was higher than that of the reported oat yogurt made by adding an aqueous extract of chickpeas (58%) [14]. It was revealed that a porous microstructure of EPS with a high molecular weight promoted the retention of a large amount of water molecules. Additionally, the network structure formed by the interaction between EPS, starch, and protein in oats might have hindered the free flow of water. As compared to the results of cohesiveness and apparent viscosity, the set-type oat yogurt showed a higher cohesiveness ratio of 0.51, compared to the 0.43 ratio of the oat paste (*p* < 0.05) (Figure 2B), as well as the top apparent viscosity curve of the set-type oat yogurt (Figure 2C). These improved properties of the set-type oat yogurt could be due to the ability of *ST15* and *LB20249* to produce large amounts of EPS during fermentation.

### 3.4. Effect of Fermentation on Quality of Set-Type Oat Yogurt

**Figure 4:**
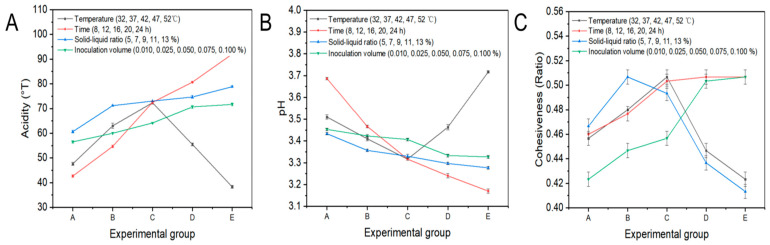
Changes in acidity (**A**), pH (**B**), and cohesiveness (**C**) of set-type oat yogurt at different temperatures, times, solid–liquid ratios, and inoculation volumes. The x-axis labels A, B, C, D, and E correspond to the variable conditions defined in the legend, in the specified order.

Sourness is a defining characteristic of yogurt (Figure 4A). As the temperature increased, the acidity gradually increased and then decreased, reaching the highest value of 72.33 °T at 42 °C (*p* < 0.05). This was due to the cultivation of lactic acid bacteria at optimal growth temperatures, which allowed them to multiply and produce acid. In addition, the acidity increased over time, reaching a maximum value of 92 °T (*p* < 0.05). However, an excessively sour taste in the set-type oat yogurt could result in an unsatisfactory sensory quality, emphasizing the importance of an optimal fermentation time. The acidity gradually increased with the solid–liquid ratio to a peak value of 78.83 °T with a maximum oat content of 13% (*p* < 0.05). This was likely attributable to the high carbohydrate and protein content in the oat flour, which acted as a carbon and nitrogen source for lactic acid bacteria, fostering the proliferation and accumulation of lactic acid [25,26]. The acidity increased with higher inoculation volumes, reaching its highest value of 71.67 °T (*p* < 0.05). However, excessively high inoculation volumes negatively impacted the taste of the yogurt [27]. Contrary to the trend in acidity changes, as the temperature increased, the pH value initially decreased and then rose, reaching the lowest pH value of 3.32 at 42 °C (Figure 4B). The variables of time, solid–liquid ratio, and inoculation volumes further reduced the pH to 3.17, 3.28, and 3.33, respectively (*p* < 0.05), when the set-type oat yogurt was incubated with 0.1% lactic acid bacteria for 24 h with a solid–liquid ratio of 13%. Cohesiveness was also critical to assess the sensory quality of set-type oat yogurt (Figure 4C). Several studies reported that the EPSs produced by lactic acid bacteria effectively improved the cohesiveness of yogurt [22]. In this study, the cohesiveness initially increased with an increase in temperature but decreased from 42 °C with the highest ratio value of 0.5 (*p* < 0.05). It was speculated that 42 °C was the optimal temperature for lactic acid bacteria, which facilitated rapid proliferation and generated substantial amounts of EPSs. Over time, the cohesiveness of the set-type oat yogurt initially increased significantly (*p* < 0.05) before stabilizing at 0.50 ratio after 16 h (*p* > 0.05), which was closely related to the EPS yield. On the one hand, the growth metabolism of the lactic acid bacteria was inhibited after 16 h because of the sharp decrease in pH levels, which prevented the EPS production [28]. On the other hand, some presence of glycohydrolases probably led to the hydrolysis of EPSs into monosaccharides during prolonged fermentation [29]. Therefore, a duration of 16 h was selected for the subsequent process. As the solid–liquid ratio increased, the cohesiveness gradually increased and then stabilized, reaching the highest ratio value of 0.50 at 7% (*p* < 0.05). That was because that oat flour was necessary for starch gel formation, and also crucial for supplying more nutrients to the lactic acid bacteria, which fostered the accumulation of EPSs. The moisture content in the system was reduced by excessive oat flour, causing a dry and cracked set-type oat yogurt, which ultimately impaired its cohesiveness. The cohesiveness also gradually increased with the inoculation volume, reaching the highest ratio value of 0.50 with a 0.10% volume (*p* < 0.05). However, the excessive acidity resulting from too much of the inoculation volume had a side effect on the cohesiveness of set-type oat yogurt, when the inoculation volume was higher than 0.1%. Therefore, it was essential to maintain the inoculation volume within an appropriate range to obtain the optimal acidity and cohesiveness.

### 3.5. Effect of Fermentation on the Rheological Properties of Set-Type Oat Yogurt

**Figure 5:**
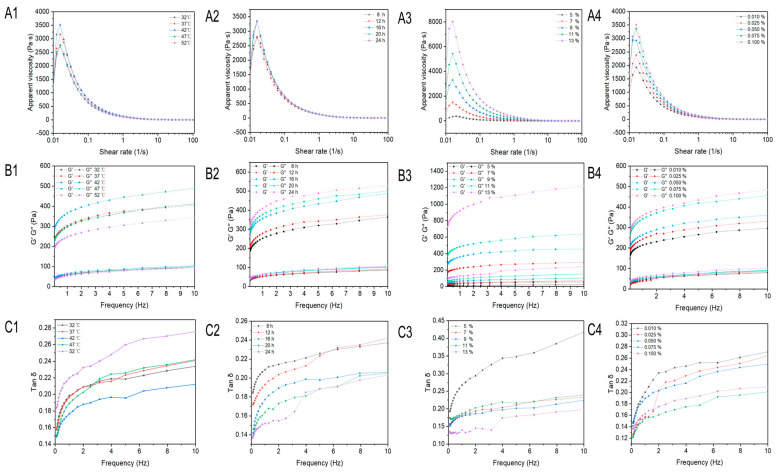
Rheological properties of set-type oat yogurt. Plots of steady shear analysis (**A**), storage modulus and loss modulus (**B**), and tan δ (G″/G′ ratio) (**C**) for set-type oat yogurt with different temperatures (1), times (2), solid–liquid ratios (3), and inoculation volumes (4).

There was a pseudoplastic, shear-thinning behavior observed in all set-type oat yogurts within non-Newtonian fluids (Figure 5). With the increase in the shear rate, the disruption rate of the intermolecular entanglements was greater than the reformation rate, thus resulting in a reduction in apparent viscosity with the set-type oat yogurt. The apparent viscosity reached the highest value of 3513 Pa·s at 42 °C, followed by the second value of 3161 Pa·s at 37 °C (*p* < 0.05), and there were no significant differences among other temperatures (Figure 5A1). Over time, the apparent viscosity reached a peak value of 3353 Pa·s at 24 h (*p* < 0.05) (Figure 5A2). With an increase in the solid–liquid ratio, the apparent viscosity achieved a maximum of 8015 Pa·s at a ratio of 13% (*p* < 0.05) (Figure 5A3). Additionally, the apparent viscosity reached its highest value of 3510 Pa·s at an inoculation volume of 0.1% (*p* < 0.05) (Figure 5A4). The increased production of EPSs by *ST15* and *LB20249* might be responsible for the higher apparent viscosity of the set-type oat yogurt. Nikitina et al. [22] also reported an increase in viscosity of the dairy product after fermentation by *Lactiplantibacillus plantarum AG10*. Additionally, the rheological results also showed that the thickening effect of oat starch could promote the increase in apparent viscosity.

The values of the storage modulus (G′) and loss modulus (G″) of the set-type oat yogurt were increased with an increase in the angular frequency, suggesting a frequency dependency (Figure 5B1–B4). The G′ of set-type oat yogurt was much higher than G″, implying that the set-type oat yogurt was still dominated by elasticity [30]. The temperature caused an initial increase in the values of G′ and G″, but a decrease occurred when it was higher than 42 °C (Figure 5B1). Both the G′ and G″ values of the set-type oat yogurt were increased by a longer fermentation duration, reaching the highest values at 24 h (Figure 5B2). A much more significant increase in the values of G′ and G″ of the set-type oat yogurt was induced by a high solid–liquid ratio, namely a higher oat flour percentage (Figure 5B3). As the inoculation volume increased, the G′ and G″ values of the set-type oat yogurt rose correspondingly (Figure 5B4). This was probably because of the EPS generation, which further promoted the interactions between EPSs and oat starch, consequently modifying the gel network structure. In addition, the tan δ value of the oat yogurt was far less than 1, further proving “solid-like” characteristics (Figure 5C1–C4). Since the reduction in the tan δ value meant an increase in the rigidity of the oat yogurt, the increase in time, inoculation volume, and solid–liquid ratio led to a strengthened oat yogurt gel, but the temperature should be appropriate, around 42 °C. These changes in rheological properties showed that the thickening effect of oat starch and its correlation with EPSs could promote the enhancement in the set-type oat yogurt gel. It indicated that EPSs played the key role in constructing the gel structure for the set-type oat yogurt. The EPS has been also reported to enhance the gel stiffness of animal yogurt by Brüls et al. [21].

### 3.6. Optimization of Acidity and Cohesiveness of Set-Type Oat Yogurt by RSM

In order to enhance the coagulated texture and sensory quality of the set-type oat yogurt, the BBD was thus employed to obtain optimal conditions of fermentation. Based on the single-factor-test data, the experiment was designed as shown in Table 3. A quadratic regression analysis was conducted to evaluate the relationships between the independent and response variables by establishing an empirical model. As expected, four models were obtained and are shown in Equations (4)–(7).

Y_1_ = 67.50 + 3.64A + 5.15B − 2.31C + 1.82AB + 1.00AC − 1.62BC − 1.25A^2^ − 3.18B^2^ − 13.65C^2^ (4)

Y_2_ = 3.36 − 0.0437A − 0.0600B + 0.0288C − 0.0225AB − 0.0100AC + 0.0125BC + 0.0085A^2^ + 0.0260B^2^ + 0.1435C^2^ (5)

Y_3_ = 0.5060 + 0.0050A + 0.0100B − 0.0075C + 0.0075AB + 0.0025AC − 0.0125BC − 0.0017A^2^ − 0.0267B^2^ − 0.0418C^2^ (6)

Y_4_ = 1315.80 + 100.16A + 1327.11B − 112.83C + 70.82AB − 68.85AC − 67.45BC − 131.31A^2^ + 536.54B^2^ − 252.09C^2^ (7)

**Figure 6:**
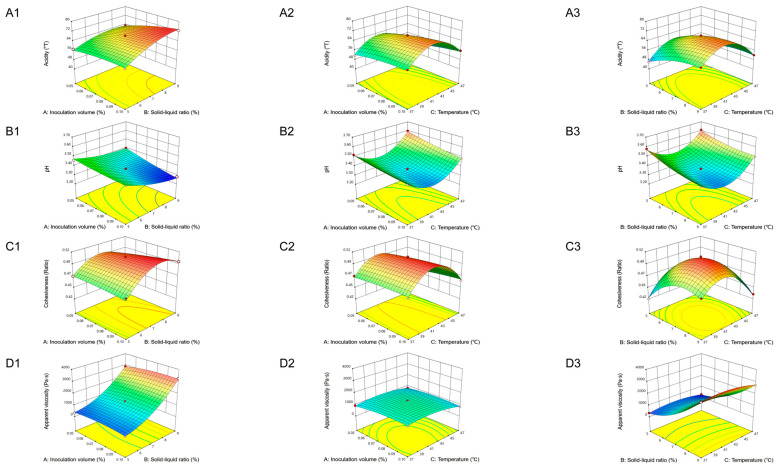
Response surface and contour plots for acidity (**A**), pH (**B**), cohesiveness (**C**), and apparent viscosity (**D**). 1, inoculation volume and solid–liquid ratio; 2, inoculation volume and temperature; 3, solid–liquid ratio and temperature. Red color means the higher response value (acidity, pH, cohesiveness, and apparent viscosity), while green/blue color means the lower response value (acidity, pH, cohesiveness, and apparent viscosity).

Y_1_, Y_2_, Y_3_, and Y_4_ represent the quadratic models for acidity, pH, cohesiveness, and apparent viscosity in coded variables. A, B, and C represent inoculation volume (%), solid–liquid ratio (%), and temperature (°C), respectively. All equations were significant at the 95% confidence level due to their *p*-values being less than 0.05 (Table 4 and Tables S1–S3). The determination coefficients (R^2^) of equations were 0.9930, 0.9902, 0.9838, and 0.9992, respectively, indicating that only 0.7%, 0.98%, 1.62%, and 0.08% of the total variations were not explained by each model. The insignificant lack of fit (0.3448, 0.3387, 0.4428, and 0.1303; *p* > 0.05) indicated that all models were adequate under the selected experimental conditions [31]. The 3D response surfaces constructed with the help of the experimental design are shown in Figure 6. Overall, the optimal production conditions for the set-type oat yogurt using the RSM model were an inoculation volume of 0.1%, a solid–liquid ratio of 7.6%, and a temperature of 41.6 °C. In order to simplify the production, the solid–liquid ratio and temperature were adjusted to 8% and 42 °C, respectively. The experimental optimization was validated with an acidity of 73.17 °T, a pH of 3.21, a cohesiveness ratio of 0.51, and an apparent viscosity of 1902.67 Pa·s, all of which are closely aligned with the RSM results. The acidity of plant-based yogurt has been reported, such as chickpea yogurt (32.42 °T), fava bean yogurt (34.82 °T), and pea yogurt (30.49 °T) [32]. The oat yogurt produced by adding a 3% aqueous extract of chickpeas was also reported, and its pH value was 4.20 [14]. The cohesiveness of chickpea yogurt (0.38), fava bean yogurt (0.39), and pea yogurt (0.44) was reported by Qin et al. [32]. The viscosity of oat yogurt combined with potato protein and potato starch was reported by Greis et al. (10 Pa·s) [33]. Additionally, the EPS production by the optimal combination of *ST15* and *LB20249* was 304.99 mg/L, which was much higher than the reported EPS yield of 42.9 mg/L by *Streptococcus thermophilus T9* only [34]. In comparison, the set-type oat yogurt in this study showed higher acidity, cohesiveness, viscosity values, a lower pH value, as well as more EPSs. This indicated that the optimal fermentation conditions in this study effectively promoted the production of lactic acid bacteria.

### 3.7. Taste Attributes of Optimal Set-Type Oat Yogurt

**Figure 7:**
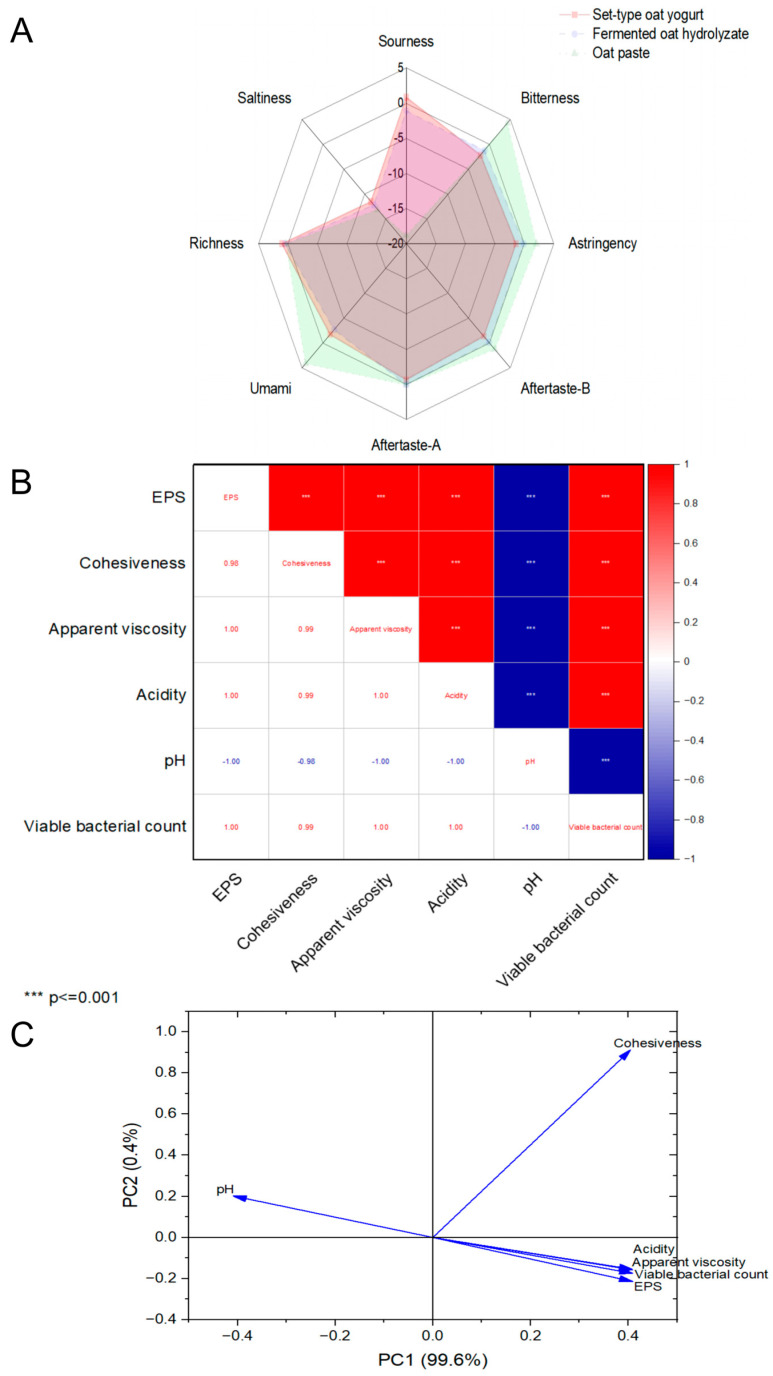
Spider plot for electronic tongue sensory score of fermented oat hydrolysate, oat paste, and set-type oat yogurt (**A**). Correlation analysis of EPS and set-type oat yogurt cohesiveness, apparent viscosity, acidity, pH, and viable bacterial count (**B**). Principal component analysis (PCA) of EPS and set-type oat yogurt cohesiveness, apparent viscosity, acidity, pH, and viable bacterial count (**C**).

The taste attributes were able to provide a taste “fingerprint” of complex liquid or semisolid samples, including sourness, bitterness, astringency, umami, richness, saltiness, aftertaste-A, and aftertaste-B. In comparison to fermented oat hydrolysate and oat paste, the set-type oat yogurt demonstrated the highest values for sourness, saltiness, and richness, while exhibiting the lowest values for bitterness, astringency, aftertaste-A, and aftertaste-B (Figure 7A). This indicated that the optimal processing of the set-type oat yogurt exhibited the most favorable taste profile.

### 3.8. The Key Role of EPSs in Set-Type Oat Yogurt Quality

The textural and rheological properties of the set-type oat yogurt reflected the microstructure changes in the process of fermentation [35]. The acidity, pH, and viable bacterial count of the set-type oat yogurt directly determined the satisfaction of consumers. The Pearson correlation coefficient was used to analyze the correlations of the set-type oat yogurt quality with its internal material base (Figure 7B). The positive correlation of the EPS content with both the cohesiveness and apparent viscosity of the set-type oat yogurt was especially significant. Comparable observations have also been previously reported; the cohesiveness and apparent viscosity of the yogurt containing EPSs were higher than those of the control group [22]. This finding was due to the ability of EPSs to strengthen the three-dimensional network structure within the set-type yogurt. Additionally, the acidity and viable bacterial count of the set-type oat yogurt were positively correlated with the EPS content, whereas the pH value showed a significant negative correlation with the EPS content (*p* < 0.001). This was attributed to the quantity and activity of fermentative bacteria, which in turn promoted the EPS generation in the set-type oat yogurt. The result of the PCA also supported the close correlations among EPS yield, cohesiveness, apparent viscosity, acidity, pH, and viable bacterial count (Figure 7C). Principal components 1 and 2 accounted for 99.6% and 0.4% of the total variance, respectively. A positive correlation was observed between the EPS yield and the cohesiveness, apparent viscosity, acidity, and viable bacterial count in the set-type oat yogurt. This finding further illustrated the key role of the EPS yield in the satisfactory quality of the set-type oat yogurt.

## 4. Conclusions

This study investigated the development of a novel set-type oat yogurt through the fermentation of a mixture of oat enzymatic hydrolysate and whole oat flour. The oat hydrolysate provided sufficient nutrients for the growth of lactic acid bacteria. The combination of lactic acid bacteria strains *ST15* and *LB20249* significantly accelerated the fermentation process and promoted the coagulated texture of the set-type oat yogurt. Therefore, the desirable acidity, pH, cohesiveness, and apparent viscosity values of the set-type oat yogurt were obtained, which improve the sensory quality. The improvement was also supported by its superior sourness, saltiness, and richness. Most importantly, these improvements were attributed to the EPS production, which contributed to the “solid-like” characteristics and subsequent coagulated texture according to the rheological properties results and PCA analysis. In summary, this study innovatively solved the technical problems of set-type oat yogurt, including the lack of fermentable carbon sources, phase separation, poor coagulation, and undesirable texture. It fills the gap in the industry and demonstrates substantial market application potential. The study highlights the potential of oat hydrolysate, EPSs, and oat flour in enhancing the sensory attributes or coagulated texture of set-type oat yogurt. Further research is required to elucidate the exact interactions of EPSs with oat starch or protein and their impact on the textural properties of set-type oat yogurt.

## Figures and Tables

**Table 1:** Experimental and predicted response for the dextrose equivalent (DE) value of oat enzymatic hydrolysate during the liquefaction process using an orthogonal design.

Serial Number	A: Enzyme Concentration	B: Temperature	C: Time	D: pH	DE Value
1	1	1	1	1	37.67
2	2	1	2	2	40.76
3	3	1	3	3	41.69
4	2	2	1	3	42.73
5	3	2	2	1	40.16
6	1	2	3	2	41.01
7	3	3	1	2	37.47
8	1	3	2	3	37.94
9	2	3	3	1	37.79
K1	116.62	120.12	117.87	115.62	
K2	121.28	123.90	118.86	119.24	
K3	119.32	113.20	120.49	122.36	
K avg1	38.87	40.04	39.29	38.54	
K avg2	40.43	41.30	39.62	39.75	
K avg3	39.77	37.73	40.16	40.79	
Optimal level	A_2_	B_2_	C_3_	D_3_	
Best combination	A_2_B_2_C_3_D_3_				

**Table 2:** Experimental and predicted response for the DE value of oat enzymatic hydrolysate during the saccharification process using an orthogonal design.

Serial Number	A: Enzyme Concentration	B: Temperature	C: Time	D: pH	DE Value
1	1	1	1	1	81.02
2	2	1	2	2	84.61
3	3	1	3	3	82.95
4	2	2	1	3	87.92
5	3	2	2	1	87.11
6	1	2	3	2	87.45
7	3	3	1	2	81.86
8	1	3	2	3	80.07
9	2	3	3	1	82.79
K1	250.47	252.07	253.42	252.36	
K2	256.02	262.97	252.49	256.29	
K3	254.45	245.90	255.03	252.29	
K avg1	83.49	84.02	84.47	84.12	
K avg2	85.34	87.66	84.16	85.43	
K avg3	84.82	81.97	85.01	84.10	
Optimal level	A2	B2	C3	D2	
Best combination	A_2_B_2_C_3_D_2_				

**Table 3:** Box–Behnken design matrix with experimental runs and observed responses.

Runs	A: Inoculation Volume (%)	B: Solid–Liquid Ratio (%)	C: Temperature (°C)	Acidity (°T)	pH	Cohesiveness (Ratio)	Apparent Viscosity (Pa·s)
1	0.100	7	37	58.20	3.44	0.47	1202.0
2	0.100	7	47	55.70	3.48	0.46	825.1
3	0.050	9	42	63.20	3.40	0.47	2884.0
4	0.075	7	42	68.60	3.37	0.50	1273.0
5	0.050	7	47	45.00	3.60	0.45	800.5
6	0.100	9	42	72.70	3.28	0.50	3264.0
7	0.075	5	37	46.00	3.58	0.42	337.6
8	0.075	5	47	44.50	3.61	0.43	260.4
9	0.075	7	42	67.10	3.35	0.51	1328.
10	0.050	7	37	51.50	3.52	0.47	902.0
11	0.075	7	42	66.60	3.36	0.50	1300.0
12	0.075	7	42	66.60	3.37	0.51	1338.0
13	0.100	5	42	59.30	3.43	0.47	416.4
14	0.050	5	42	57.10	3.46	0.47	319.7
15	0.075	7	42	68.60	3.34	0.51	1340.0
16	0.075	9	47	52.10	3.50	0.43	2728.0
17	0.075	9	37	60.10	3.42	0.47	3075.0

**Table 4:** Analysis of variance for the fitted quadratic polynomial model with acidity as the response value.

Source	Sum of Squares	Degrees of Freedom	Mean Square	F-Value	*p*-Value	Significance
Model	1257.43	9	139.71	109.92	<0.0001	Significant
A	105.85	1	105.85	83.28	<0.0001	
B	212.18	1	212.18	166.93	<0.0001	
C	42.78	1	42.78	33.66	0.0007	
AB	13.32	1	13.32	10.48	0.0143	
AC	4.00	1	4.00	3.15	0.1193	
BC	10.56	1	10.56	8.31	0.0236	
A^2^	6.58	1	6.58	5.18	0.0571	
B^2^	42.44	1	42.44	33.39	0.0007	
C^2^	784.52	1	784.52	617.21	<0.0001	
Residual	8.90	7	1.27			
Lack of fit	4.70	3	1.57	1.49	0.3448	Not significant
Pure error	4.20	4	1.05			
Cor total	1266.33	16				
R^2^	0.9930		Std. Dev.	1.13		
Adj R^2^	0.9839		Mean	58.99		
Pred R^2^	0.9355		C.V. %	1.91		
Adeqprecision	33.3646		Press	81.72		

## Data Availability

The original contributions presented in the study are included in the article/Supplementary Material, further inquiries can be directed to the corresponding author.

## Supplementary Materials

The following supporting information can be downloaded at: https://www.mdpi.com/article/10.3390/foods13244180/s1. Table S1: Analysis of Variance analysis for the fitted quadratic polynomial model with pH as the response value; Table S2: Analysis of Variance analysis for the fitted quadratic polynomial model with cohesiveness as the response value; Table S3: Analysis of Variance analysis for the fitted quadratic polynomial model with apparent viscosity as the response value.
