# Supplementary material for: Optimization of Enzymatic Hydrolysis and Fermentation Processing for Set-Type Oat Yogurt with Favorable Acidity and Coagulated Texture
Source: Foods. 2024 Dec 23;13(24):4180. doi: 10.3390/foods13244180 (PMC11675732; doi:10.3390/foods13244180)
Supplement: Supplementary file 1 [file foods-13-04180-s001.zip › foods-3341211-supplementary.pdf]

Supplementary Materials:

Table S1-3 showed the coefficients of the models calculated using ANOVA analysis.

Table S1. Analysis of Variance analysis for the fitted quadratic polynomial model with pH as the response value.

Source	Sum of squares	Degrees of freedom	Mean square	F-value	P-value	Significance
Model	0.1466	9	0.0163	78.36	< 0.0001	Significant
A	0.0153	1	0.0153	73.67	< 0.0001	
B	0.0288	1	0.0288	138.56	< 0.0001	
C	0.0066	1	0.0066	31.81	0.0008	
AB	0.0020	1	0.0020	9.74	0.0168	
AC	0.0004	1	0.0004	1.92	0.2079	
BC	0.0006	1	0.0006	3.01	0.1265	
A^2	0.0003	1	0.0003	1.46	0.2656	
B^2	0.0028	1	0.0028	13.69	0.0077	
C^2	0.0867	1	0.0867	417.13	< 0.0001	
Residual	0.0015	7	0.0002			
Lack of fit	0.0008	3	0.0003	1.52	0.3387	Not significant
Pure error	0.0007	4	0.0002			
Cor total	0.1480	16				
R^2	0.9902		Std. Dev.	0.0144		
Adj R^2	0.9775		Mean	3.44		
Pred R^2	0.9091		C.V. %	0.4189		
Adeq precision	30.5222		Press	0.0135		

Table S2. Analysis of Variance analysis for the fitted quadratic polynomial model with cohesiveness as the response value.

Source	Sum of squares	Degrees of freedom	Mean square	F-value	P-value	Significance
Model	0.0133	9	0.0015	47.14	< 0.0001	Significant
A	0.0002	1	0.0002	6.36	0.0397	
B	0.0008	1	0.0008	25.45	0.0015	
C	0.0004	1	0.0004	14.32	0.0069	
AB	0.0002	1	0.0002	7.16	0.0317	
AC	0.0000	1	0.0000	0.7955	0.4021	
BC	0.0006	1	0.0006	19.89	0.0029	
A^2	0.0000	1	0.0000	0.4103	0.5422	
B^2	0.0030	1	0.0030	95.86	< 0.0001	
C^2	0.0073	1	0.0073	233.52	< 0.0001	
Residual	0.0002	7	0.0000			
Lack of fit	0.0001	3	0.0000	1.11	0.4428	Not significant
Pure error	0.0001	4	0.0000			
Cor total	0.0136	16				
R^2	0.9838		Std. Dev.	0.0056		
Adj R^2	0.9629		Mean	0.4729		
Pred R^2	0.8681		C.V. %	1.19		
Adeq precision	19.4200		Press	0.0018		

Table S3. Analysis of Variance analysis for the fitted quadratic polynomial model with apparent viscosity as the response value.

Source	Sum of squares	Degrees of freedom	Mean square	F-value	P-value	Significance
Model	1.58E+07	9	1.76E+06	1031.66	< 0.0001	Significant
A	80260.21	1	80260.21	47.14	0.0002	
B	1.41E+07	1	1.41E+07	8275.09	< 0.0001	
C	1.02E+05	1	1.02E+05	59.81	0.0001	
AB	20064.72	1	20064.72	11.78	0.0109	
AC	18961.29	1	18961.29	11.14	0.0125	
BC	18198.01	1	18198.01	10.69	0.0137	
A^2	72601.99	1	72601.99	42.64	0.0003	
B^2	1.21E+06	1	1.21E+06	711.87	< 0.0001	
C^2	2.68E+05	1	2.68E+05	157.15	< 0.0001	
Residual	11918.76	7	1702.68			
Lack of fit	8609.96	3	2869.99	3.47	0.1303	Not significant
Pure error	3308.8	4	827.2			
Cor total	1.58E+07	16				
R^2	0.9992		Std. Dev.	41.26		
Adj R^2	0.9983		Mean	1387.86		
Pred R^2	0.991		C.V. %	2.97		
Adeq precision	94.5207		Press	1.43E+05		
